# Characterization of burn wound healing gel prepared from human amniotic membrane and *Aloe vera* extract

**DOI:** 10.1186/s12906-019-2525-5

**Published:** 2019-06-03

**Authors:** Md Shaifur Rahman, Rashedul Islam, Md Masud Rana, Lucas-Sebastian Spitzhorn, Mohammad Shahedur Rahman, James Adjaye, Sikder M. Asaduzzaman

**Affiliations:** 1 Institute for Stem Cell Research and Regenerative Medicine, Medical Faculty, Heinrich Heine University, 40225 Düsseldorf, Germany; 2 Bio-resource Technology and Industrial Biotechnology Laboratory, Department of Biotechnology and Genetic Engineering, Jahangirnagar University, Dhaka, 1342 Bangladesh; 3 Institute of Tissue Banking and Biomaterial Research, Atomic Energy Research Establishment, Dhaka, 1349 Bangladesh

**Keywords:** Amniotic membrane, *Aloe vera*, Angiogenesis, Burn, Epithelialization, Gel, Healing, Inflammation, Wound

## Abstract

**Background:**

Skin burn wound is a notable medical burden worldwide. Rapid and effective treatment of burnt skin is vital to fasten wound closure and healing properly. Amniotic graft and *Aloe vera* are widely used as wound managing biomaterials. Sophisticated processing, high cost, availability, and the requirement of medics for transplantation limit the application of amnion grafts. We aim to prepare a novel gel from amnion combined with the *Aloe vera* extract for burn wound healing which overcome the limitations of graft.

**Methods:**

Two percent human amniotic membrane (AM), *Aloe vera* (AV) and AM+AV gels were prepared. In vitro cytotoxicity, biocompatibility, cell attachment, proliferation, wound healing scratch assays were performed in presence of the distinct gels. After skin irritation study, second-degree burns were induced on dorsal region of Wistar rats; and gels were applied to observe the healing potential in vivo. Besides, macroscopical measurement of wound contraction and re-epithelialization; gel treated skin was histologically investigated by Hematoxylin and eosin (H&E) staining. Finally, quantitative assessment of angiogenesis, inflammation, and epithelialization was done.

**Results:**

The gels were tested to be non-cytotoxic to nauplii and compatible with human blood and skin cells. Media containing 500 μg/mL AM+AV gel were observed to promote HaCaT and HFF1 cells attachment and proliferation. In vitro scratch assay demonstrated that AM+AV significantly accelerated wound closure through migration of HaCaT cells. No erythema and edema were observed in skin irritation experiments confirming the applicability of the gels. AV and AM+AV groups showed significantly accelerated wound closure through re-epithelialization and wound contraction with *P* < 0.01. Macroscopically, AM and AM+AV treated wound recovery rates were 87 and 90% respectively with *P* < 0.05. Histology analysis revealed significant epitheliazation and angiogenesis in AM+AV treated rats compared to control (*P* < 0.05). AM+AV treated wounds had thicker regenerated epidermis, increased number of blood vessels, and greater number of proliferating keratinocytes within the epidermis.

**Conclusion:**

We demonstrated that a gel consisting of a combination of amnion and *Aloe vera* extract has high efficacy as a burn wound healing product. Amniotic membrane combined with the carrier *Aloe vera* in gel format is easy to produce and to apply.

## Background

Globally, burn injury is rated as the fourth frequent out of all injuries. Approximately 500,000 burn patients have to be treated in the USA each year [1]. Despite recent advances in burn wound skin management, the death rates remain high throughout the world [2] with a vast majority (eleven fold higher) in low-income countries [3]. For instance, in 2003, about 173,000 children were burned in Bangladesh where burn is the fifth leading cause of childhood illness [4]. However, in particular, peoples of rural areas are more prone to the burden of burn related mortalities and morbidities. Fast aid and swift treatment for burn patients are vital to increase the survivability by closuring and protecting the burn wounds as immediately as possible to mitigate disabilities and fatalities. In most of the developing countries, peoples would like to treat burn injuries immediately at home before going to a clinic [3]. Nevertheless, there are still lacking of a low cost and effective fast aid product to manage of burn injuries in an efficient and rapid manner [5].

Pathophysiologically, burn is considered one of the most severe types of wound as it is easily susceptible to infection due to vascular necrotic tissue and loosening of epidermal integrity [6]. Healing of wounds is a dynamic process including various overlapping phases [7] such as ‘early inflammatory phase’ that inhibits infection during healing as well as destroys necrotic tissue and triggers signals essential for wound repair [8], the ‘proliferative phase’ involves wound closure and restoration of vascular network [9] and finally the wound scar matures during the ‘wound remodeling phase’ [10]. Nonetheless, burn wound healing is often interrupted by excess inflammation leading to delayed healing and increased pain. Furthermore, scar tissue formation, incomplete re-epithelialization and absence of complete collagen remodeling are also hindering issues of burn healing [11, 12]. During skin wound healing two cell types, namely keratinocytes and fibroblasts, interact in the proliferative phase [9]. By a feedback loop keratinocytes and fibroblasts increase cell proliferation rate and wound contraction vigorously [13]. The immune cell- macrophages that stimulate keratinocytes and fibroblasts to release the factors for increasing angiogenesis, collagen production, and epithelialization [7]. Due to having these features, keratinocytes based burn wound healing products such as single cell keratinocyte spray solution and keratinocyte cell sheet are available in the developed world [13, 14]. In 2013, the possibility of amniotic fluid derived mesenchymal cells (AF-MSCs) as a source for cell based wound healing therapy has been reported [15, 16]. But the usefulness of these highly sophisticated and costly therapeutic products remains out of affordability for third world people.

Human amniotic membrane graft is one of the most medically accepted and widely used biomaterials in burn wound healing treatment from 1910 on [17, 18]. It acts as a scaffold for proliferation and differentiation of new epithelial cells due to presence of factors such as fibronectin, elastin, nidogen, collagen types I, III, IV, V, VI, and hyaluronic acid [19–21]. Alongside lacking of histocompatibility antigens HLA-A, B and DR [22], it possesses an anti-inflammatory effect [23]. However, the processing, transportation and storage of intact thin sheet of amniotic membrane has limited clinical applications due to associated cost. In 2017, the Atala group reported that dissolved amniotic membrane with hyaluronic acid gel can speed up the skin wound healing process [24]. Besides the human materials, plants extract are also experimented to have burn healing properties. For instance, *Aloe vera* (AV) has been used in treating burn associated wound and observed to be effective in burn wound management [25, 26]. Because of anti-inflammatory effects, AV is of high usefulness in the treatment of skin wounds and first to second degree burns [27]. Additionally, AV treatment significantly increased the collagen synthesis and remodels collagen composition (type III) to promote wound healing, contraction and the breaking strength of resulting scar tissue [28, 29]. Importantly, it has been demonstrated that AV has greater efficacy over silver sulfadiazine cream in the treatment of second-degree burns [30, 31]. In the developed world, recombinant growth factors and cellular tissue-engineered skin substitutes-based wound treatments are available and clinically practiced [32]. However, this sophisticated approach is associated with high costs for patients in the low-income countries [33]. Some reported commercial skin grafts such as integra and biobrane are available which have been shown to improve wound healing but they are also expensive and sometimes do not deliver optimal outcomes [34, 35]. Thus, there is a need for a wound healing product with high clinical efficiency, which can be used rapidly, but retains the activity of a biological treatment.

Clinically, amnion has been applied as a wound covering bioactive material to heal split thickness skin burn wounds as well as for children with partial-thickness facial burns [36, 37]. From our experience, amniotic membrane as a graft for burn wounds enclosure in Bangladesh appears to be advantageous [38]. But the limited number of membrane donors and the lack of trained personnel in amniotic graft processing are major challenges. Further, amniotic membrane grafting service is available only in city areas at a very limited scale. Other limitations including instant requirement of physicians to do the transplantation of the graft and the sheet of amnion is generally held in place with sutures or additional bandaging [24]. Considering the described treatment limitations on the one side and the advantages of wound healing properties of human amnion and *Aloe vera* on the other side; this study aimed to develop a novel cost efficient product which in fact should be easy to produce and to store, physiologically effective and which application does not require a medic. Thus, we prepared three novel gel products from the extract of amniotic membrane (AM), *Aloe vera* (AV) and the combination (AM+AV) which were later characterized both in vitro and in vivo*.* We have demonstrated the usefulness of AM, AV and AM+AV gels as wound healing biomaterials which can accelerate burn wound closure through contraction, re-epithelialization, reduced inflammation and increasing angiogenesis in an animal model for skin burn.

## Methods

### Ethical approval

The collection and use of cesarean sections derived amniotic membrane for research and grafting purpose was approved by the ethical committee of Atomic Energy Research Establishment, and permitted by the “Human Organ / Tissue Donation and Transplantation Act, 1999” Govt. of Bangladesh. Written consent from the amniotic membrane donor was taken for amniotic membrane collection for use in research purpose. The ethics committee of Jahangirnagar University recommended and approved the animal model (Wistar Rats) for this study of skin irritation, burn induction following ARRIVE guidelines. All efforts were made to prevent any unnecessary and harmful animal handling.

### Collection of placenta and preparation of human amniotic membrane

Human placenta/amniotic sacs were collected during cesarean sections and kept in 4 °C. Within 24 h we processed and prepared the membrane as described before [24, 38]. Amniotic membranes were separated carefully from chorionic membrane manually and washed with PBS (Gibco) repeatedly until a complete elimination of blood clots was achieved. After that, the whitish membranes were transferred into sterile petri dishes and frozen at subzero temperature overnight and freeze-dried (Alpha1-4LD, CHRIST, Germany) at − 55 °C for 24 h. Dried amniotic membranes were sterilized using gamma radiation at 10KGy with cobalt-60γ radiation sources. The membranes were then aseptically processed into powder form which was later used for gel preparation.

### Preparation of *Aloe vera* extract

Fresh *Aloe vera* leaves were collected from the medicinal plant garden of NIB (National Institute of Biotechnology), Bangladesh by Rashedul Islam and Md Masud Rana. The plant *Aloe vera* were identified and collection of leaves kindly permitted by Md Moniruzzaman, Scientific Officer, NIB, Bangladesh. First of all, the leaves were washed with distilled water (DW) and wiped with 70% ethanol. The lower part of the leaves was cut to allow *Aloe* latex to be removed. After removal of latex, the leaves were taken into a laminar chamber and cut in equal pieces of about 4cm^2^. The leaves were merged in absolute alcohol for 5 min to further sterilize. Leaves were then peeled off and the juice was collected by scraping. Afterwards, the juice was poured into dishes and allowed to freeze; and finally freeze dried at − 55 °C for 48 h to obtain powder form. It was possible to extract 635 g juice from 1 kg of leaves which finally led to 8 g of powder.

### Formulation and evaluation of physico-chemical properties of gel

From the dried amniotic membrane powder and *Aloe vera* powder, 2 gram of each sample was used for gel preparation. In total three types of gel formulations were prepared (i) AM (6% CMC-Na (Loba Chemie), 2% AM (2 g of amniotic membrane powder), 0.02% methyl paraben (SUPELCO-Sigma Aldrich), 5% glycerine (CP, China), 0.05% triethanol-amine (Merck), and DW up to 100 ml), (ii) AV (6% CMC-Na, 2% AV (2 g of *Aloe vera* powder), 0.02% methyl paraben, 5% glycerine, 0.05% triethanol-amine, and DW up to 100 ml), and (iii) AM+AV (6% CMC-Na, 1% AM (1 g of amniotic membrane powder), 1% AV (1 g of *Aloe vera* powder), 0.02% methyl paraben, 5% glycerine, 0.05% triethanol-amine, and DW up to 100 ml). The homogeneity of all formulated gels was confirmed by visual analysis. For assessing the pH of the different gel preparations, 2.5 g of each gel was dissolved in 25 ml of DW and incubated for 2 h. The measurements were done in triplicates and the average values were taken into consideration. The pH of these gels ranged from 6.5 to 6.9.

### In vitro biocompatibility and cytotoxicity assay

In vitro biocompatibility and cytotoxicity test were done as described by Khan et al., (2012) [39]. To do the heparinized human blood biocompatibility assay, AM, AV and AM+AV gels were diluted with different ratios of blood. A blood sample of the same donor diluted with DW and saline water (SW) at the same ratios as done for the gels was used as a control. After an incubation time of 2 h at room temperature, the blood/gel mixtures were spread on glass slides, and observed under a light microscope for possible morphological changes in the blood cells.

In vitro cytotoxicity tests of the AM, AV and AM+AV gels were performed using the brine shrimp (*Artemia salina*) lethality bioassay method. *Artemia salina* eggs were hatched in a 1 L conical flask, filled with sterile artificial sea water (pH = 8.5) and constant aeration for 48 h. After hatching, active nauplii free from egg shells were collected and used for the assay. All three gels were dissolved in artificial seawater at 2.0, 1.0, 0.75, 0.50, and 0.25 mg/mL concentration in petri dishes in which the active nauplii were inoculated. After overnight incubation, the viability of the nauplii was counted. Artificial sea water without additions served as negative control and 0.50 mg/mL of vincristine sulfate (Sigma) was considered as positive control.

Human skin cell biocompatibility tests were performed by exposing HaCaT cells (CLS Heidelberg, Germany) for 48 h in the specific culture medium containing the formulated gels at distinct concentrations. Cells were cultured in DMEM (Gibco/Life Technologies) with 10% Fetal Bovine Serum (FBS) (Gibco/Life Technologies) and 1% Penicillin/Streptomycin (Gibco) at 37 °C in 5%CO_2._ These tests resulted in an optimal gel concentration of 500 μg/mL.

### In vitro cell attachment and proliferating assay

For cell attachment study, 50 thousand of human keratinocytes (HaCaT) and human fibroblast (HFF1 cell line (ATCC; SCRC-1041)) cells were seeded in 2 ml media containing the previously prepared gels at a concentration of 500 μg/mL. The cells were allowed to attach to the culture dish undisturbed for 2, 4, 6, and 8 h in case of HFF1; for 3, 6, 9 and 12 h in case of HaCaT, respectively. At each time interval microscopic images were taken to evaluate the attachment rate of the cells in the different conditions. To assess the proliferation of HaCaT and HFF1, equal numbers of cells were expanded on plates in the media containing AM, AV and AM+AV gels. The images were taken from day 2 to day 6 to visualize the proliferation of the cells. Media were replaced every other day.

### In vitro wound healing scratch assay

The scratch assay was performed as described by D’Agostino and co-workers to study cell migration and to determine the time period required for wound closure in vitro [40] in presence of the three gel formulations (500 mg/mL). When cells attained 95–100% confluency, HaCaT and HFF1 were serum starved for 24 h before initiation of the scratch wound. Scratch wounds were created in confluent cell monolayers using a sterile p200 pipette tip ensuring that each wound had the same dimensions. After that, the cells which were detached by this process were removed from the culture dish by three times washing with PBS. Cell migration and wound closure were observed at 0 h, 18 h and 30 h and images were taken by light microscopy.

### In vivo irritability study

To assess in vivo irritability and applicability of the gels, the dorsal skin hairs of female Wistar rats were shaved on the date of experiment [41]. Total 12 animals were experimented and randomly were assigned to three groups (AM, AV and AM+AV). The animals were treated with 1 ml gel daily up to 7 days and finally the treated skin was visually examined for erythema and edema.

### Rat model for artificial burn induction, re-epithelization and wound contraction

In total 40 healthy female Wistar rats of 180–200 g body weight were used in this study and randomly assigned into four experimental groups (control/no gel, treated with AV, treated with AM and treated with AM+AV). All animals received human care according to the guideline for the care and use of laboratory animals published by NIH. Rats were fed a standard rat chow and tap water ad libitum. The rats were kept in the animal quarter at a temperature of 25 ± 2 °C, humidity 50–55% and with 14 h light/10 h dark cycles. Each rat was anesthetized with Ketamine HCl solution (Gonoshasthaya Pharmaceuticals Ltd., Bangladesh) of 100 mg/Kg body weight by intra peritoneal injection. Subsequently, the hair at dorsal region was trimmed using electric hair clipper and then shaved with sharp blade. The shaved areas were cleansed with alcohol swab.

Burns were created using a piece of aluminum (981.875mm^2^) heated to 100 °C for 5mins which was applied for 15 s on the shaved area of rats [42]. The animals were treated with 1 ml of the distinct gel on daily basis, topically, for a period of 30 days. Re-epithelization was monitored by recording the number of days required for crust to fall away, leaving no raw wound behind [43]. To monitor wound contraction, progressive changes in wound area were measured. Using the formula [42] below, the percentage of wound contraction was calculated on the respective day.

$$ \%\kern0.5em Wound\kern0.5em Contraction=\left(\left( Initial\kern0.5em Wound\kern0.5em Size- Final\kern0.5em Wound\kern0.5em Size\right)/ Initial\kern0.5em Wound\kern0.5em Size\right)\times 100 $$

At the specific days, the gel treated and non-treated animals were anesthetized by intraperitoneally administration of 100 mg/kg ketamine, skin tissue/biopsy were taken and finally rats were sacrificed by cervical dislocation. Skin tissues were collected for histopathological analysis.

### Histology of skin: hematoxylin and eosin (H&E) staining

Skin specimens from each group were collected on the 6th, 12th, 18th, 24th, and 30th day after burn induction. Skin biopsies were embedded in paraffin blocks after overnight fixation in 10% formal saline solution. Embedded skin tissues were cut into sections of 5 μm thickness using a microtome (Leica, RM 2125 RTS, USA) and collected on glass slides. Afterwards, the sections were deparaffinized and stained with H&E. The stained histological sections were examined and evaluated in random order. Images were taken with Optika B-350 light microscope. A score of 0–3 was given to each section according to presence of inflammatory cells and levels of angiogenesis and epithelialization as previously described by Sedighi et al.*,* 2016 [44] and Kulac et al.*,* 2013 [45] with minimal modifications (Table 1).

**Table 1:** Histological scoring parameter of epithelialization, angiogenesis, granulation tissue formation, and inflammatory cells

Parameter/Score	0	1	2	3
Inflammatory cells	1–5 inflammatory cells per histological field	5–8 inflammatory cells per histological field	8–11 inflammatory cells per histological field	11–15 inflammatory cells per histological field
Epithelialization	Absence of epithelial proliferation in ≥70% of tissue	Incomplete epidermal organization in ≥50% of tissue	Moderate epithelial proliferation in ≥60% of tissue	Complete epidermal remodeling in ≥80% of tissue
Angiogenesis	Absence of angiogenesis including congestion and hemorrhage	2–4 vessel per site, congestion and hemorrhage	4–6 vessel per site, slight congestion	7–8 vessel per site vertically disposed towards the epithelial surface
Granulation Tissue	None, completely disorganized and distorted	Minimal/immature thin	Mild/moderately mature granule layer	Evident/ Thick, ≥80% organized

### Statistical analysis

All statistical analyses were calculated by one way independent test using SPSS (SPSS version 22.0, SPSS Inc., Chicago, IL, USA). All quantitative data were presented in this study including mean (±) standard deviations (SD). *P* < 0.05 was considered as statistically significant.

## Results

### Preparation and physico-chemical properties of gel

**Fig. 1:**
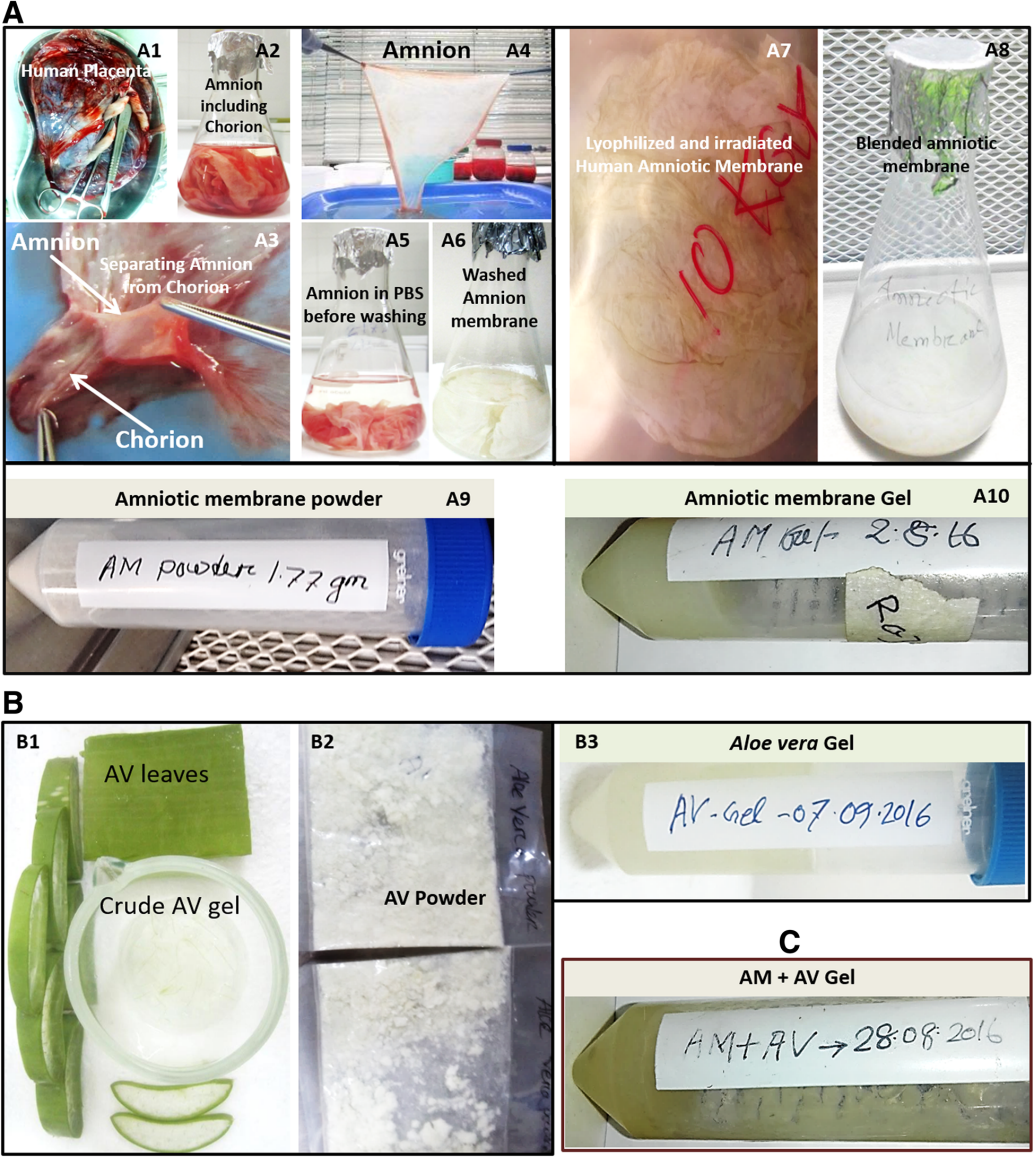
Processing of amnion membrane and *Aloe vera* and physical appearance of the formulated gels. **a** Preparation of amniotic membrane from human placenta (a1, a2, a3, a4, a5 and a6). The dried lyophilized amniotic membrane was 10KGy gamma irradiated and blended. Then the freeze-dried powder was used to formulate AM gel (a7, a8, a9 and a10). **b** *Aloe vera* leaves (b1) and freeze-dried *Aloe vera* extracted powder (b2). The formulated gels AM, AV and AM + AV were shown in a10, a3, and **c** respectively

Donated full term human placentas were collected during cesarean sections, the erythrocytes were depleted and the amnion was separated (Fig. 1a1-a6) for further processing. Afterwards, amniotic membranes were lyophilized, gamma irradiated, blended and finally freeze-dried to obtain powder (Fig. 1a7-a9). The juice of fresh *Aloe vera* leaves was collected and freeze-dried to extract powder for further use (Fig. 1b1-b2).

The obtained amnion (AM) and *Aloe vera* (AV) powders were used to formulate the 2% gels (AM, AV and AM+AV) as described in methods section. The pH of all three gel formulations was measured and ranged from 6.5 to 6.9 (AM 6.7, AV 6.5 and AM+AV 6.9). All formulated gels were found to be semi-solid and homogeneous in nature. The color of the AV gel was off-white while the AM and AM+AV gels were yellowish white whereas all gels were semi-transparent (Fig. 1a10, b3, c).

### In vitro biocompatibility and cytotoxicity analysis

**Fig. 2:**
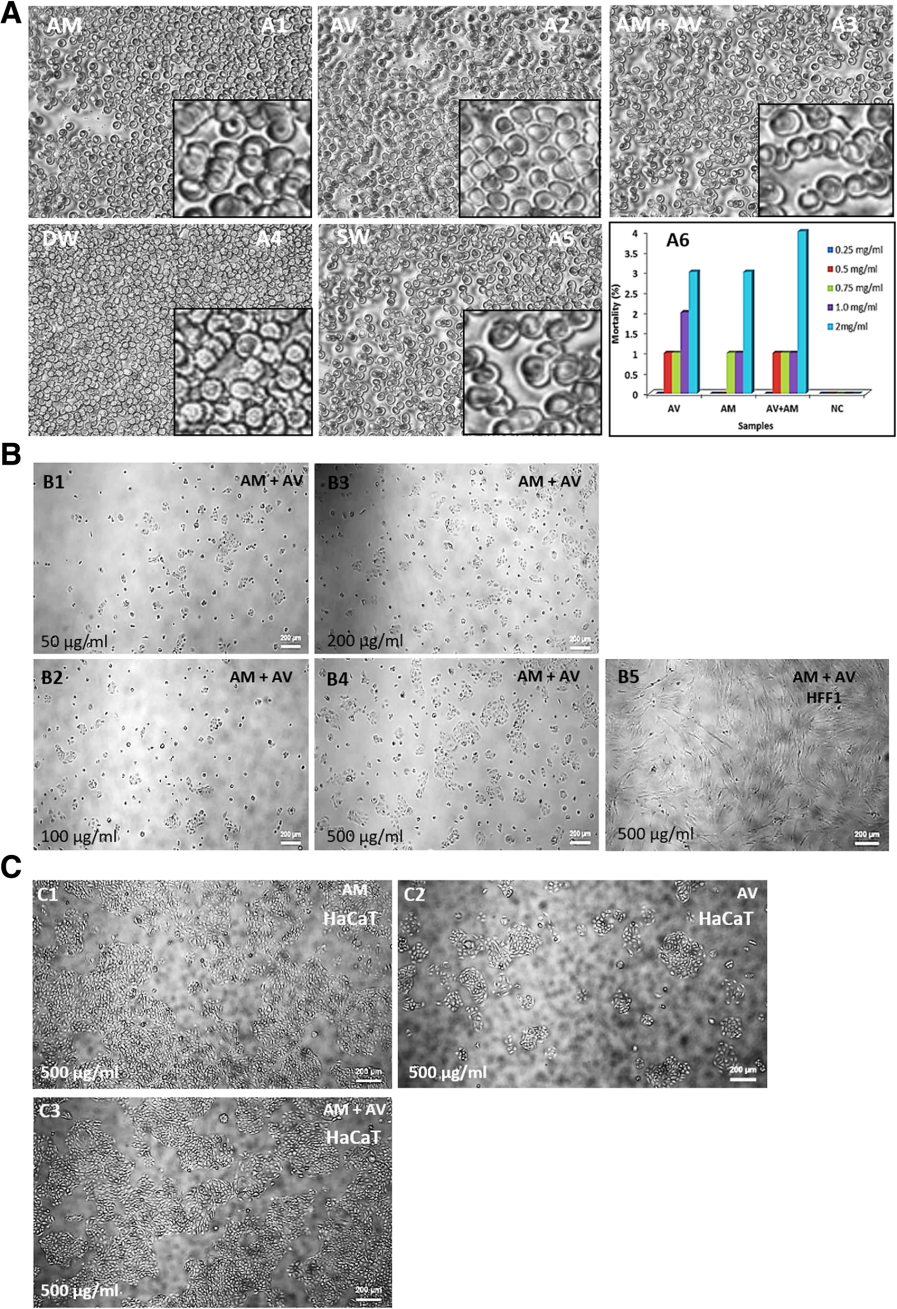
Heparinized human blood biocompatibility (**a**), brine shrimp lethality bioassay (a6); HaCaT and HFF1 cells compatibility with gels (**b** and **c**). RBC morphology of heparinized blood incubated with AM (a1), AV (a2), AM+AV (a3), DW (a4) and SW (a5). a6 diagram shows the mortality percentages of brine shrimp (*A. salina*). 500 μg/mL was shown to be suitable for HaCaT and HFF1 cells in vitro (**b** and **c**)

Upon incubation of human blood with gel samples (2:1 ratio), red blood cells (RBCs) were observed to be intact (Fig. 2a1-a3). Normal saline (SW) also showed similar results (Fig. 2a5) whereas DW caused RBC lysis (Fig. 2a4).

Brine shrimp lethality bioassay method was used to evaluate the in vitro cytotoxic effect of the formulated AM, AV and AM+AV gels (Fig. 2a6). A concentration of 0.25 mg/ml did not affect the viability. At a concentration of 0.5 mg/ml and 0.75 mg/ml, the mortality rate of the nauplii was 1%. Increasing the concentration (2 mg/ml) of gels led to an increase in mortality rate to 3–4% (Fig. 2a6).

Keratinocytes (HaCaT) and fibroblasts (HFF1) play important roles in the skin tissue during wound healing processes. Therefore, we tested biocompatibility of AM+AV gels on HaCaT cells at concentrations of 50-500 μg/mL (Fig. 2b1-b4) and could not observe any difference in cell viability. The concentration of 500 μg/mL was also observed to be compatible for human fetal foreskin derived fibroblast (HFF1) cells when cultured in presence of AM+AV (Fig. 2b5). Together with the previous results we observed 500 μg /mL concentration (Fig. 2b4) as an optimum gel concentration for in vitro use which was further confirmed for the compatibility of AV as well as AM gels (Fig. 2c1-c3).

### Attachment and proliferation of HaCaT and HFF1 cells in vitro

**Fig. 3:**
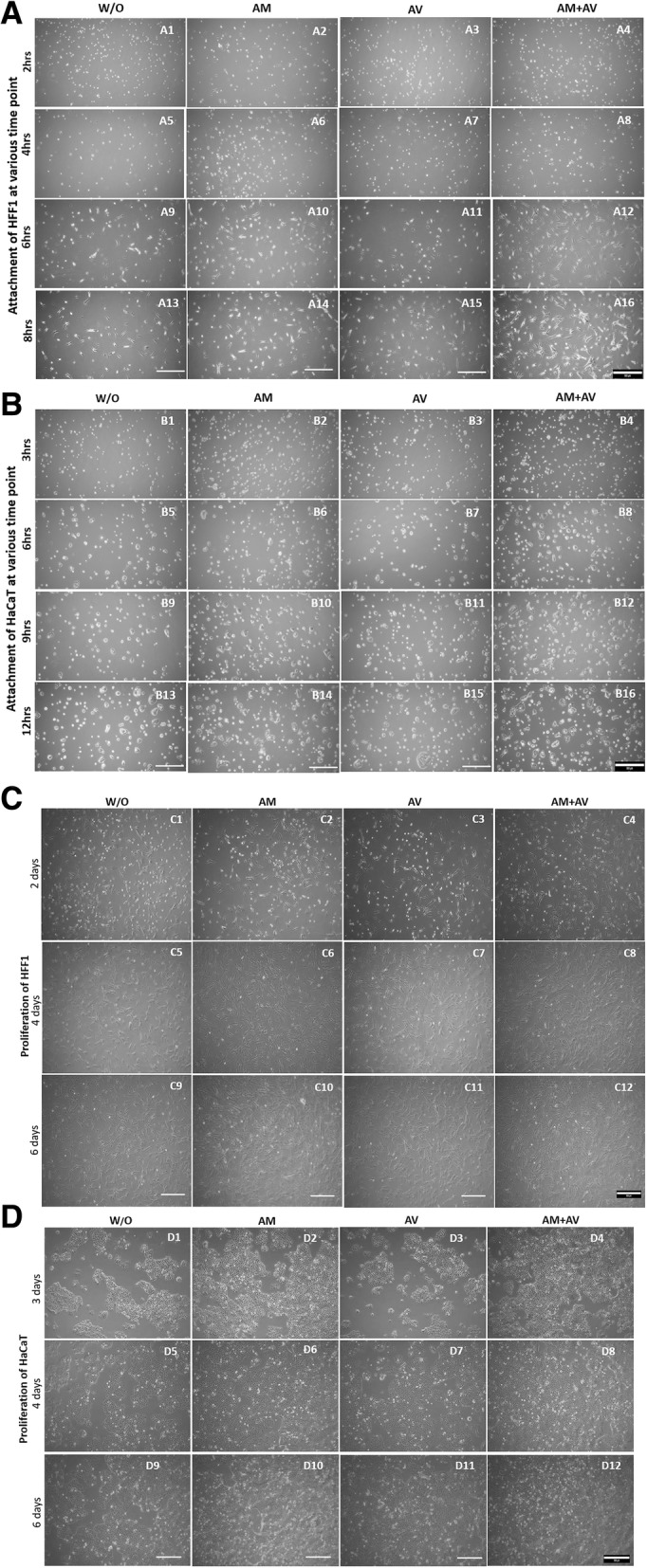
Representative microscopic images for HFF1 and HaCaT cell attachment and proliferation. **a** HFF1 cells (2 h to 8 h) and (**b**) HaCaT cells (3 h to 12 h) attachment test. **c** Proliferation tests of HFF1 for 2, 4, and 6 days. **d** The proliferation of HaCaT from day 3 to day 6 in media with and without gels

The time required for the attachment of HaCaT and HFF1 in presence of the three different types of gel (AM, AV, AM+AV) was evaluated. No effects on HFF1 cell attachment within the first 2 h were noted in presence of AV and AM gels compared to control media (Fig. 3a1-a3). But HFF1 in AM+AV gel containing media showed a better attachment rate within the first 2 h (Fig. 3a4). At six to 8 hours of culture duration, it was visually observed that the HFF1 attachment rate in the AM+AV condition was approximately 1.5 fold higher (Fig. 3a12, a16) than in the other conditions.

However, HaCaT cells took at least 3 h to attach on culture dish in presence of the gels (AM, AV, AM+AV) in media at the concentration of 500 μg/mL (Fig. 3b1-b4). The HaCaT cell attachment rates were significantly higher when we applied AM and AM+AV gel in the culture media (Fig. 3b2, b6, b10; Fig. 3b4, b8, b12). In case of AV, we did not observe any difference between media alone and media with AV at various time points (Fig. 3b1, b3; Fig. 3b5, b7; Fig. 3b9, b11; Fig. 3b13, b15). Beside the cell attachment study, we also qualitatively examined the proliferation of HFF1 and HaCaT when incubated with the gels from day two to day six. We did not observe any significant effect of the gels on the proliferation of HFF1 cells during the examination periods (Fig. 3c1-c12). However, media with AM and AV + AM gels were noticed to increase the proliferation rate of HaCaT cells two fold (Fig. 3d1-d12).

### In vitro wound healing and in vivo irritability study of the gels

**Fig. 4:**
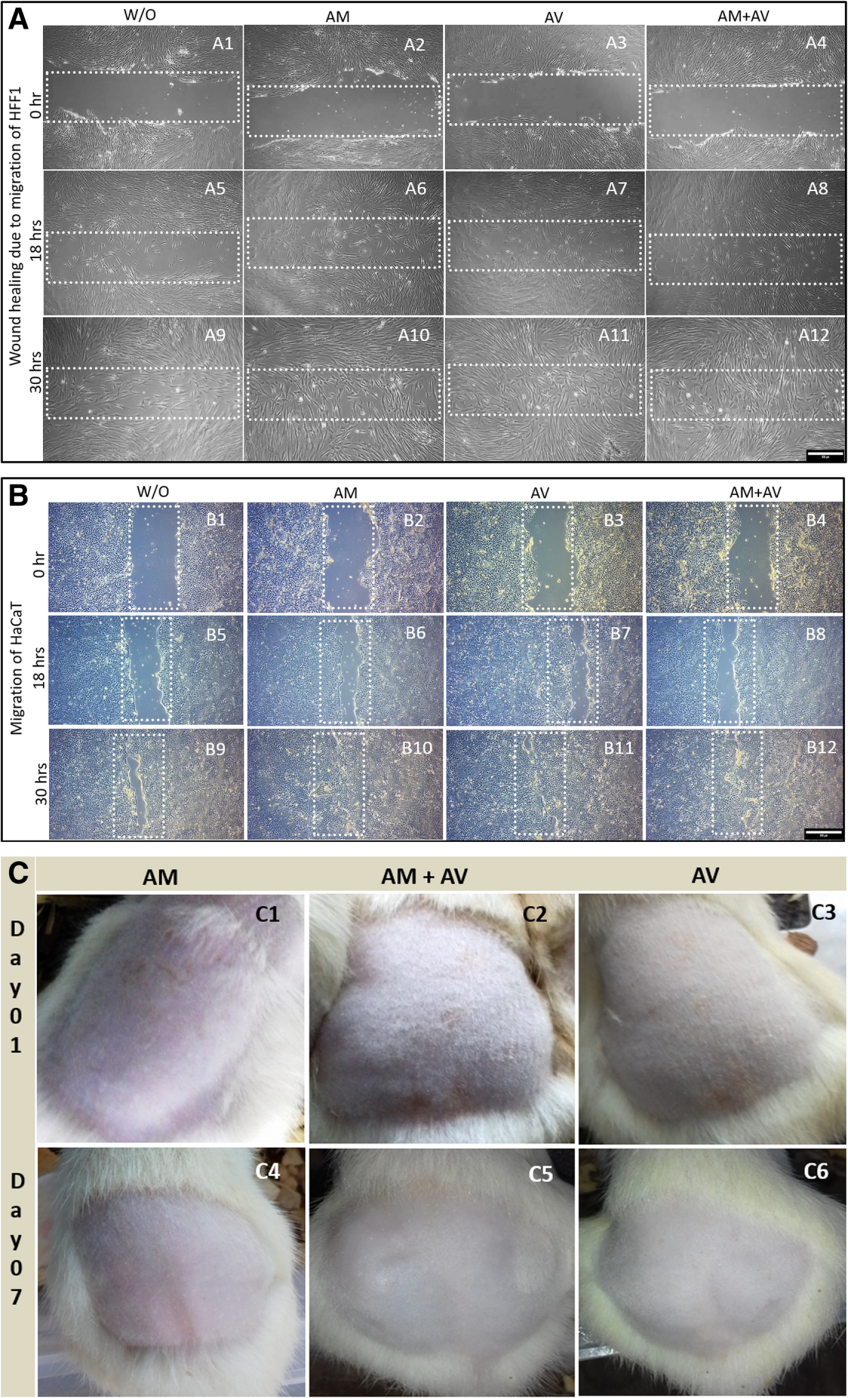
In vitro wound healing and in vivo skin irritation study. **a** Migration of HFF1 cells to close the wound area. **b** The HaCaT cell migration to fill the scratch zone in presence of the three different gels in the cell culture media whereas cell culture medium without gels served as the negative control. **c** Topical application of the gels for irritation assay on shaved rats, which were observed from day 0 to day 7

The in vitro scratch assay was performed to measure cell migration in the scratching zone. Consequently, we wanted to detect whether these formulated gels (concentration 500 μg/mL) can promote the rate of wound healing in human keratinocytes (HaCaT) and fibroblasts (HFF1) via scratch assays. The cells were serum starved for at least 24 h before producing the scratch wound. Thus, the cells’ ability to proliferate was inhibited, and it was assured that wound closure was only due to cell migration. Our result showed that the healing velocity of HaCaT and HFF1 with AM and AM+AV gel treatment was higher than for untreated cells and AV treated (Fig. 4a, b). HaCaT cells filled the scratch area faster when compared to HFF1 after 30 h.

To analyze the applicability and irritability of the prepared gel, skin irritation assay were performed applying a rat model. After topical application of all gels for a period of 7 days, it was observed that the gel did not induce any edema or erythema (Fig. 4c). This result indicated the safety of gels to be applied topically. We also observed that the hair formation was also normal compared to non-treated rats.

### Macroscopic evaluation of wound closure and quantitative measurement of wound contraction and re-epithelialization

**Fig. 5:**
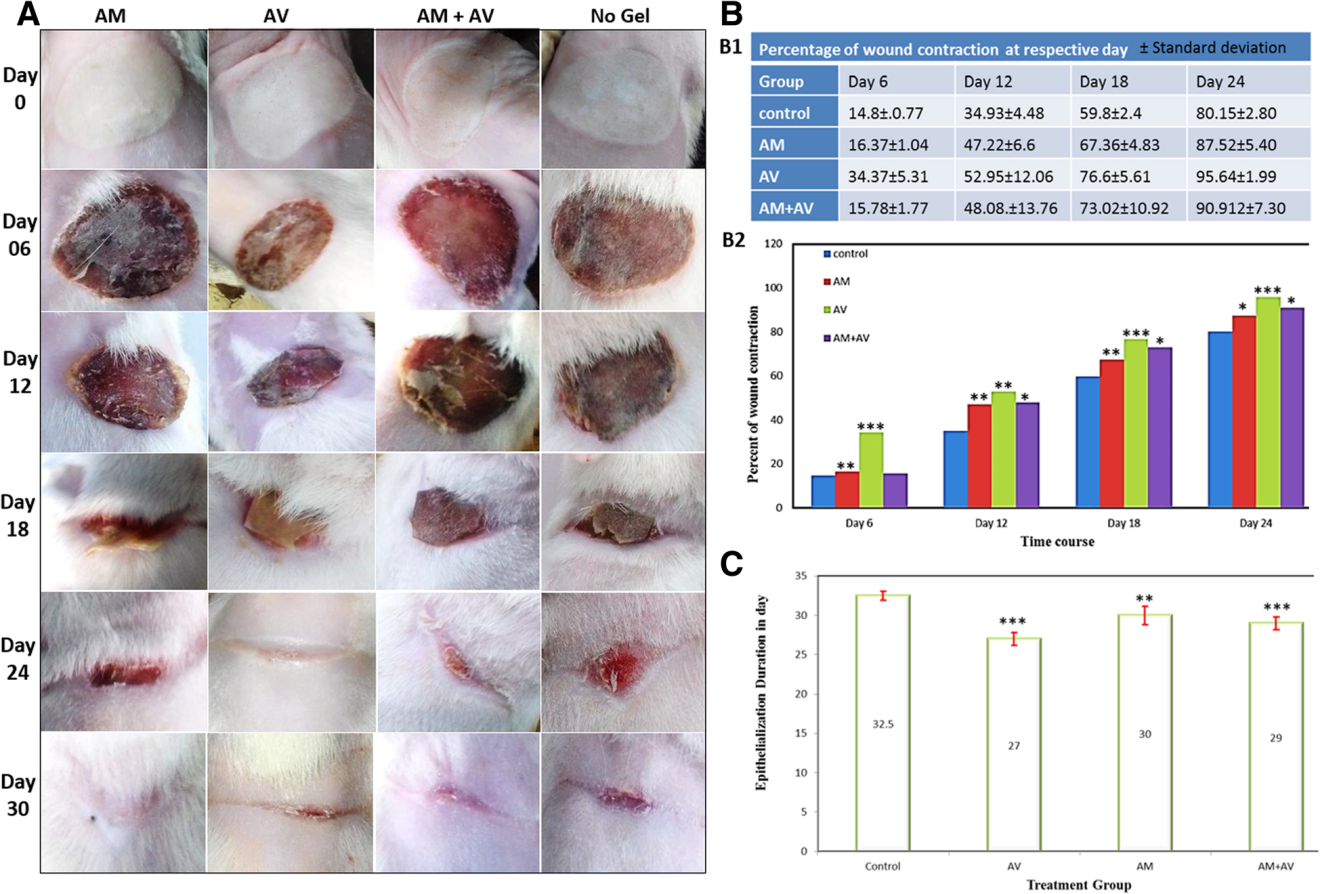
Evaluation of wound closure, measurement of wound contraction and re-epithelialization. **a** Macroscopic wound healing images of Wistar rats at day 0, 6, 12, 18, 24 and 30 which were treated with AM, AV, AM+AV gel or without any gel (control). **b** Clinical evaluation of wound healing regarding wound contraction in rats at day 6, 12, 18, 24 (b1) and (b2) (level of significant was set * P < 0.05, ** P < 0.01 and *** P < 0.001). **c** Epithelialization period in different groups (*P < 0.05, ** P < 0.01 and *** P < 0.001)

After few hours of the second degree burn induction, the rats were restless indicating the pain. No bubbles were observed on the burn area, however, it was noticed that white color burn damaged the skin barrier. Subsequently, hyperemia occurred into the damaged tissue area. After treating the rats with gels at day one, within few hours, white color burn turned into a fully hyperemic zone in each group which indicated the presence of red blood cells undergoing extravasation (Fig. 5a, day 0 panel). At day six, all groups showed the presence of thick dry crusts but the group treated with AM+AV gel had a slightly wet crust (Fig. 5a day 6 panel). Edges of crust were found to be partially detached. At 12 days after burn induction, control group showed discrete detachment of crust while other treatment groups showed continuous detachment of edges. In this stage of injury staining of crusts were almost same. As the full burn wound was covered with crust scar tissue was not observed until the 12th day (Fig. 5a, day 12 panel). On day 18, scar tissue became clearly visible in the edges of the wounds in each group and contractions were clearly visible (Fig. 5a, day 18 panel). At day 24, crusts disappeared from all groups and scar tissue became clearly visible. The AV treated group showed faster healing but this group left more scar tissue than AM and control groups (Fig. 5a, day 24 panel). Re-epithelialization had been completed in all treatment groups leaving scar tissues after 4 weeks (Fig. 5a, day 30 panel). Macroscopically, the AV group showed a better healing rate but included more scar tissue while other groups showed less healing rate than the AV group with minimal scar formation.

However, a variation in wound contraction rates were noticed from group to group (Fig. 5b1, b2). At day 6, the AV treated group showed significantly (*P* < 0.001) better healing rate which was two folds higher than in the other groups. The healing rate of the AM treated group was also found to be significantly increased (*P* < 0.01) when compared to AM+AV (*P* > 0.05). Twelve days post burning, the control group had the lowest healing rate (34.93%) whereas AV treated group wound healing was about 50% (*P* < 0.01). At the same time point, the healing rate of the AM treated group was 47.22% (*P* < 0.01) and of the AM+AV 48.08% of the wound were found to be healed (*P* < 0.05) (Fig. 5b1, b2). On the 18th day, AV treated group reached a healing rate of 76.6% (*P* < 0.001). It was observed that the wound healing rate of the AM+AV group (73%) was better than in the AM treated group (67%). But, statistical evaluation showed a higher significance in the AM treated group (*P* < 0.01) in comparison with AM+AV (*P* < 0.05). At day 24, the percent of wound contraction in the AV group also showed better results compared with the other two groups. Interestingly, wounds from the AV group were demonstrated to be healed about 95% (*P* < 0.001) whereas the control group showed a healing of 80%. Nevertheless, AM treated and AM+AV treated wound recovery rate were 87 and 90% respectively with *P* < 0.05 (Fig. 5b1, b2).

Average re-epithelialization period in all groups were observed (Fig. 5c). In AV treated animals mean epithelialization was visualized within 27 days of post burning, control group required at least 32.5 days. The re-epithelialization period of AM and AM+AV treated rats took 30 and 29 days, respectively. However, both AV and AM+AV groups were statistically significant (*P* < 0.001) (Fig. 5c).

### Histological analysis in terms of skin tissue organization, angiogenesis, and re-epithelialization

**Fig. 6:**
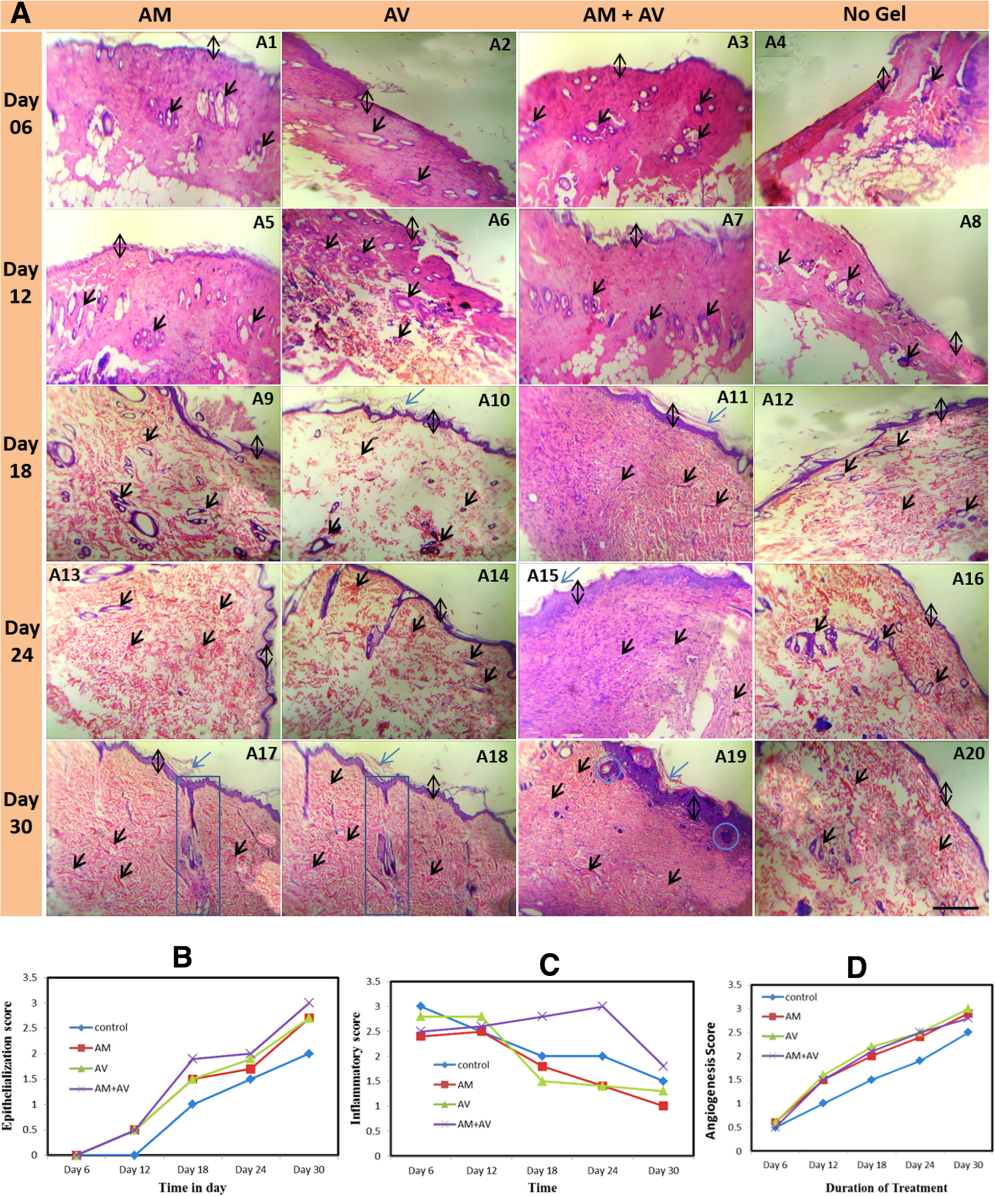
Haematoxylin and Eosin (H&E) staining of skin at different time points during the gel-based treatment (**a**). Stained images (100x magnification) were shown for all four groups (AM, AV, AM+AV, and non-treated control) at day 6 (a1-a4), 12 (a5-a8), 18 (a9-a12), 24 (a13-a16) and 30 (a17-a20). Re-epithelialization (**b**), inflammatory (**c**), and angiogenesis (**d**) scoring from the images represents a qualitative and quantitative assessment of healing. Black double headed arrow represents the thickness of epidermis; single headed blue arrow shows the keratinized zone of skin, single headed black arrow projects the blood vessel, formation of new glands beneath the epidermis is shown in blue box (a17, a18), and inside the blue round box (a19) some basal keratinocytes were shown

Figure 6a shows representative photomicrograph of skin sections stained with H&E from all groups. The comparison of angiogenesis, inflammation score and re-epithelialization along with treatment duration between all experimental groups are presented in Fig. 6b, c and d. At day 6, coagulative necrosis was identified including damage of dermal and epidermal layer (Fig. 6a1-a4). Remarkably, inflammatory cells in the wound area were increased in all groups. Highest infiltrations of inflammatory cells were detected in non-treated rats, and AM treated animals showed the lowest infiltration (Fig. 6c). In the AV and AM+AV group moderate numbers of inflammatory cells were observed (Fig. 6a3, a4).

Within the first few days no significant difference in angiogenesis score was observed amongst the groups (Fig. 6a1-a4, 6d). However, AM and AM+AV gel treated skin were shown to have appearance of more neo-vascularization (Fig. 6a5-a20) with time. On the 12th day post burning, all treated groups appeared to have induced neo-epithelialization, in contrast to the control (Fig. 6a5-a8, 6b). At day 18, neo-epithelization was clearly visible with incomplete epidermal and dermal layer (Fig. 6a9-a12, 6b).

After 18 days, the number of inflammatory cells was also reduced significantly in AM and AV treated groups. But, in AM+AV treated group the inflammatory score was increased in a significant amount (*P* < 0.05); even higher than in the control group (Fig. 6c). Angiogenesis was significantly increased in each group (*P* < 0.05) at day 18 post burning (Fig. 6d) compared to control.

The H&E stained skin section of day 24 showed an increase in epithelialization level (Fig. 6a13-a16). Epithelialization score was found to be significantly increased in each treatment group compared to the control group. Among treated groups, AM+AV had a higher epithelialization (*P* < 0.05) rate than AV (Fig. 6b). However, at this stage inflammation in AM+AV treated group increased again, while other treatment groups showed reduced inflammation (Fig. 6c). At day 24, angiogenesis in the AV group (*P* < 0.05), was higher than in the AM and the AM+AV group (Fig. 6d) but all groups showed elevated levels than the control.

H&E stained skin biopsies on the 30th day of treatment displayed maximum epithelialization in the AM+AV group including more inflammatory cells (Fig. 6a17-a20, 6b, 6c). Moreover, angiogenesis was also increased in AM and AM+AV group compared to control (Fig. 6d).

## Discussion

Beside active ingredients, the applicability of burn wound healing gel is dependent on the properties such as pH, appearance, homogeneity, and viscosity. Carbopol is a widely used gelling agent for producing burn-healing gels for skin application. CMC-Na salt is another agent used alongside with carbopol [46]. However, in this study the gel was prepared from human amniotic membrane extract (AM), *Aloe vera* extracts (AV), and a combination of both AM+AV using 6% CMC-Na salt as a gelling agent. Extensive studies confirm the effectiveness and safety of lyophilized amnion as a wound dressing and grafting materials for promoting the healing process and preventing infections [47]. Khorasani et al.*,* (2009) reported that AV cream or gel could be more effective than silver sulfadiazine cream in treating burn wound healing [30]. The end products (gels) in all three conditions evaluated in this study were homogenous, granule less and of a whitish creamy color (Fig. 1a10, 1b3, 1c). In addition, pH of all formulations ranged between 6.5–6.9, and this pH does not interfere with skin physiology [48]. Erythema and edema are the common symptoms of skin irritation which lasts for three to 7 days [49]. Our formulated gels did not induce any irritation including no erythema or edema on rat skin upon topical application for a period of 7 days (Fig. 4c). Thus, CMC-Na salt, AM and AV extract formulated gel could be useful as wound healing gel as the gels have good spreadability and consistency. In vitro and in vivo studies clearly and collectively demonstrated the potentials of the formulated gels from amnion when combined with *Aloe vera*. In this preliminary research, we found that the formulated gels can induce and accelerate the proliferation and attachment of HaCaT and HFF1 cells (Fig. 3), and promote wound healing in vitro (Fig. 4a, b) [50]. Amnion has been reported to facilitate the migration of epithelial cells, reinforces attachment, and promotes proliferation [51].

Macroscopic morphological analysis demonstrated the gel-based acceleration of re-epithelialization and wound contraction in vivo (Fig. 5a, b, c). Microscopic observation of AM+AV gel treated tissue sections allowed us to appreciate the effectiveness of the formulated gel in regard to epidermis and dermis formation and the thickness of epidermis (Fig. 6a). In present study, we observed that upon completion of wound healing few scar tissues remained in all treated rats. Scar formation in all kinds of wound healing are normal [52] and exist even after complete healing. However, the AM treated experimental group had the lowest scar formation. Regardless of the variation in treatment procedures, second degree burn required 25–35 days to heal completely [53]. Due to presence of anti-inflammatory characteristics [23, 44], amniotic gel treated rats had lower inflammation than AV and AM+AV (Fig. 6c). Inflammation is an early stage event for burn healing which should be decreased within a certain healing period [54]. During wound healing, at early phases inflammatory cells increased but in later stages decreased gradually due to granulation, the formation of new capillaries, and deposition of collagen [2]. Excluding collagen level determination, other incidences are similar to our study [55]. Histological analysis revealed that the AM+AV group had a higher epithelialization rate apart from inflammation (Fig. 6a19, 6b). Wound healing is related to wound contraction and wound re-epithelialization [56] which has been shown for gel treatment in a mouse model [50].

Angiogenesis is another important event in burn healing where endothelial cells’ proliferation rate in the wound area is rapidly increased after burning to form blood vessels. Hamid and Soliman (2015) reported that AV can increase angiogenesis [2] for a better supply with nutrients and oxygen because of acemannan in AV [57]. Histologically, AM, AV and AM+AV treated wounds had increased numbers of blood vessels, particularly small and newly formed (Fig. 6a17-a19, 6d). Besides supporting in vitro keratinocyte proliferation, we also observed some proliferating basal keratinocytes in vivo residing in the intermediate zone of the epidermis and dermis (Fig. 6a19). These properties of the tested gels could be explained by the presence of stimulatory factors in amniotic membrane which also been supported from the results of Murphy et al. (2017) [24].

Amniotic membrane has been reported to provide a niche for the cells to adhere, grow, proliferate, migrate and differentiate, and could possibly contribute to the production of angiogenic micro-environment indirectly which allows AM to improve burn healing [58]. We found that a combination of both AM and AV synergistically improved epithelialization. On day 30, epithelialization profile was significantly higher in the AM+AV group. Amniotic membrane is composed of collagen type IV, V and VII which promotes growth of epithelial cells, facilitates epithelial cell migration, strengthens basal epithelial cell adhesion, promotes differentiation of epithelial cell, and prevents apoptotic cell death [59]. In principal, we have prepared gels from medically discarded materials, at low cost that provides excellent burn wound coverage. These formulated gels showed potential to be used as fast aid ointment in burn wound management. Although we demonstrated significant improvement of burn wound healing in rats treated with AM+AV gel, however, some limitations are associated with this animal model such as the remarkable native regeneration potential of the rat skin. For future application, it would be crucial to identify the key factors in the amnion that are responsible for the acceleration of the wound healing process.

## Conclusion

Taken together the in vitro and in vivo data, our findings clearly demonstrate that amniotic membrane combined with *Aloe vera* extract significantly enhances burn wound healing, thus indicating that the amniotic membrane and *Aloe vera* possesses potent wound healing activities. Amniotic membrane is a globally accepted biological biomaterial for second and third degree burn. *Aloe vera* gel has been reported to have burn healing capacity as well. The combination of both AM and AV has been shown to have promising effect in internal epithelialization with less scar formation. Gels containing AM extract individually and in combination with AV could be used alternatively in the treatment of burn. However, further investigation is required to assess optimal concentration of the used extracts and key factors present in AM and AV to find out the best combination for burn healing. Moreover, it is also of great importance to unfold the underlying molecular mechanisms. As a further investigation step, cell based therapies using skin progenitor cells in combination with the tested gels, could be another way to accelerate wound healing.

## Data Availability

The data and materials have been presented in the main manuscript and can be given upon request.

## Abbreviations

AM: Amniotic membrane
AV: *Aloe vera*
CMC-Na: Sodium carboxymethyl cellulose
C-sections: Caesarean sections
H&E: Hematoxylin and eosin
HaCaT: (Ha = human adult, Ca = calcium, T = temperature)
HCl: Hydrogen chloride
HFF1: Human foreskin fibroblast 1
HLA: Human leukocyte antigens
HLA-DR: Human leukocyte antigen – antigen D related
KGy: Kilo gray
PBS: Phosphate buffer saline
SD: Standard deviations
